# Methylotroph Infections and Chronic Granulomatous Disease

**DOI:** 10.3201/eid2203.151265

**Published:** 2016-03

**Authors:** E. Liana Falcone, Jennifer R. Petts, Mary Beth Fasano, Bradley Ford, William M. Nauseef, João Farela Neves, Maria João Simões, Millard L. Tierce, M. Teresa de la Morena, David E. Greenberg, Christa S. Zerbe, Adrian M. Zelazny, Steven M. Holland

**Affiliations:** National Institutes of Health, Bethesda, Maryland, USA (E.L. Falcone, C.S. Zerbe, A.M. Zelazny, S.M. Holland);; University of Iowa Hospital and Clinics, Iowa City, Iowa, USA (J.R. Petts, M.B. Fasano, B. Ford, W.M. Nauseef);; Veterans Administration Medical Center, Iowa City (W.M. Nauseef);; Centro Hospitalar de Lisboa Central, Lisbon, Portugal (J.F. Neves);; Instituto Nacional de Saúde Dr. Ricardo Jorge, Lisbon (M.J. Simões);; Detroit Medical Center, Detroit, Michigan, USA (M.L. Tierce IV);; University of Texas Southwestern Medical Center, Dallas, Texas, USA (M.T. de la Morena, D.E. Greenberg)

**Keywords:** chronic granulomatous disease, methylotroph, Granulibacter bethesdensis, Acidomonas methanolica, Methylobacterium lusitanum, bacteria

## Abstract

Chronic granulomatous disease (CGD) is a primary immunodeficiency caused by a defect in production of phagocyte-derived reactive oxygen species, which leads to recurrent infections with a characteristic group of pathogens not previously known to include methylotrophs. Methylotrophs are versatile environmental bacteria that can use single-carbon organic compounds as their sole source of energy; they rarely cause disease in immunocompetent persons. We have identified 12 infections with methylotrophs (5 reported here, 7 previously reported) in patients with CGD. Methylotrophs identified were *Granulibacter bethesdensis* (9 cases), *Acidomonas methanolica* (2 cases), and *Methylobacterium lusitanum* (1 case). Two patients in Europe died; the other 10, from North and Central America, recovered after prolonged courses of antimicrobial drug therapy and, for some, surgery. Methylotrophs are emerging as disease-causing organisms in patients with CGD. For all patients, sequencing of the 16S rRNA gene was required for correct diagnosis. Geographic origin of the methylotroph strain may affect clinical management and prognosis.

## Abstract 2

Disease caused by these environmental bacteria is almost exclusively limited to patients with this condition.

Chronic granulomatous disease (CGD) is a primary immunodeficiency characterized by recurrent infections of the lung, skin, lymph nodes, and liver, as well as granulomatous inflammation affecting those organs and hollow viscera. The immunodeficiency results from deficiencies in any 1 of the 5 subunits forming the NADPH (nicotinamide adenine dinucleotide phosphate) oxidase 2 (Nox2)–based complex, which leads to impaired production of reactive oxygen species in phagocytes. Defects in the Nox2 (gp91*^phox^*) enzymatic subunit (*CYBB* [cytochrome b-245, β polypeptide]) are inherited in an X-linked manner, whereas defects in subunits p47*^phox^* (*NCF1* [neutrophil cytosolic factor 1]), p22*^phox^* (*CYBA* [cytochrome b-245, α polypeptide]), p67*^phox^* (*NCF2* [neutrophil cytosolic factor 2]), and p40*^phox^* (*NCF4* [neutrophil cytosolic factor 4]) are inherited in an autosomal recessive manner (*1*,*2*).

CGD infections are often caused by a characteristic group of pathogens, including *Staphylococcus aureus*, *Serratia marcescens*, *Burkholdheria cepacia* complex, *Nocardia* spp., and *Aspergillus* spp. (*1*). However, new pathogens are emerging, and some reportedly are found almost exclusively in patients with CGD. Methylotrophs are bacteria that can use single-carbon organic compounds as their sole source of energy, the widespread availability of which makes these organisms versatile environmental inhabitants. However, they rarely cause disease in immunocompetent persons (*3*).

We previously reported 7 methylotroph infections in patients with CGD and here describe 5 more (Table). From these 12 infections, we have isolated the methylotrophs *Granulibacter bethesdensis*, *Acidomonas methanolica*, and *Methylobacterium lusitanum*. These infections were difficult to diagnose and required prolonged courses of antimicrobial drugs and sometimes surgery for complete resolution.

**Table:** Summary of methylotroph infections in patients with chronic granulomatous disease*

Patient no., reference	Patient age, y/sex	CGD genetics†	Clinical findings	Microbiological findings	Treatment‡	Outcome
1 (this study)	1/F	Autosomal recessive (*NCF2* c.304C>T; p.R102X)	Pneumonia, cervical lymphadenitis	*Methylobacterium lusitanum* (lung), *Serratia marcescens* (lymph node)	CRO/CLI/ITZ, HSCT	Recovered
2 (this study)	19/M	X-linked (*CYBB* intragenic deletion)	Necrotizing cervical lymphadenitis	*Granulibacter bethesdensis;* *Staphylococcus epidermidis*	VAN/CRO, CFD/DOX/RIF	Recovered
3 (this study)	16/M	X-linked (*CYBB* 13-exon deletion)	Meningitis	*G. bethesdensis*	MEM/CIP/AMK/DOX/TEC/VCZ, MEM/CIP/AMK/DOX/CSP/LAMB/LZD/RIF/INH/CLR	Died
4 (this study)	9/M	X-linked (***CYBB*** point mutation; exon 10)	Cervical abscess, lymphadenitis	*Acidomonas methanolica*	TZP/VAN/LAMB/CIP, MEM/VAN/LAMB/CIP, CIP/VCZ/TMPSMX/ IFN-γ, HSCT	Recovered
5 (this study)	36/M	X-linked (*CYBB* c.1139 G>A; p.W380X)	Multifocal lymphadenitis	*G. bethesdensis;* *S. epidermidis*	VAN/CRO, CRO/DOX/TMPSMX/ITZ	Still receiving treatment as of 2015
6 (*4*)	10/M	X-linked (*CYBB* p.Arg226X)	Necrotizing cervical lymphadenitis	*A. methanolica*	TMPSMX/CRO/DOX, TMPSMX/RFB/GEN	Recovered
7 (*5*)	10/M	X-linked	Bacteremia	*G. bethesdensis*	TMPSMX/CAZ/MTZ/LZD/VCZ	Died
8 (*6*)	39/M	X-linked	Necrotizing cervical, mediastinal, axillary lymphadenitis	*G. bethesdensis*	MEM/DOX, CRO/DOX	
9 (*6*)	36/M	X-linked	Multifocal necrotizing lymphadenitis, splenic lesions, ascites	*G. bethesdensis*	MEM/TMPSMX/ITZ, MEM/TMPSMX/VCZ/TOB, CRO/TMPSMX/IFN-γ, CPD/DOX/TMPSMX/IFN-γ, splenectomy/TGC	Recovered
10 (*6*)	13/M	X-linked	Necrotizing thoracic lymphadenitis	*G. bethesdensis*, *S. epidermidis*, *Candida glabrata*, *Streptococcus mitis group*	MEM/VCZ, CRO/TOB/DOX/VCZ, CRO, DOX, CRO, CFD	Recovered
11 (*6*)	17/M	X-linked	Necrotizing cervical and mediastinal lymphadenitis	*G. bethesdensis*	LVX, DOX, lymph node excision	Recovered
12 (*6*)	37/M	X-linked	Necrotizing supraclavicular lymphadenitis, splenic and liver lesions	*G. bethesdensis*	CRO/GEN/VAN, CRO/DOX/TMPSMX, CFD/ DOX/TMPSMX, DOX/TMPSMX	Recovered

*AMK, amikacin; CAZ, ceftazidime; CFD, cefdinir; CGD, chronic granulomatous disease; CLI, clindamycin; CLR, clarithromycin; CIP, ciprofloxacin; CPD, cefpodoxime; CRO, ceftriaxone; CSP, caspofungin; *CYBB*, cytochrome b-245, β polypeptide; DOX, doxycycline; GEN, gentamicin; HSCT, hematopoietic stem cell transplant; IFN-**γ**, interferon- γ; INH, isoniazid; ITZ, itraconazole; LAMB, liposomal amphotericin B; LVX, levofloxacin; LZD, linezolid; MEM, meropenem; MTZ, metronidazole; *NCF2*, neutrophil cytosolic factor 2**; **RFB, rifabutin; RIF, rifampin; TEC, teicoplanin; TGC, tigecycline; TMPSMX, trimethoprim/sulfamethoxazole; TOB, tobramycin; VAN, vancomycin; VRZ, voriconazole. †Information in parentheses indicates, when known, the mutation that led to CGD. ‡Slashes separate drugs in the same regimen; commas separate regimens.

## Patient 1

In 2008, a 1-year-old girl from Mexico who had p67*^phox^*-deficient CGD was examined for fever, weight loss, and enlarged cervical lymph nodes; she had been receiving ceftriaxone, clindamycin, itraconazole, and interferon-γ for treatment of CGD. CGD was diagnosed when she was 6 months of age, at which time she had *Penicillium* sp. pneumonia and an abnormal dihydrorhodamine oxidation assay result. At 3 weeks of age, she had had a methicillin-sensitive *S. aureus* labial abscess, followed at 8 months of age by 3 episodes of pneumonia and an *S. marcescens* buttock abscess.

A computed tomographic (CT) scan performed at the time of admission showed cervical, mediastinal, and mesenteric lymphadenopathy and a right middle lung lobe infiltrate and hepatosplenomegaly. Excisional cervical lymph node and lung biopsy samples were processed for bacterial, nocardial, fungal, and mycobacterial cultures and staining. Direct Gram staining revealed moderate mononuclear cells but no organisms. After 11 days of incubation on chocolate agar at 37°C, the lung biopsy culture grew 1 pink colony. Species level identification conducted by full 16S rRNA gene sequencing (≈1,500 bp) showed a 99.8% match to the *M. lusitanum* type strain (*7*). Etest (bioMérieux Diagnostics, Marcy l’Etoile, France) showed the following MICs (in μg/mL): amikacin (MIC = 4), cefepime (MIC = 8), ceftriaxone (MIC = 2), ciprofloxacin (MIC = 16), piperacillin-tazobactam (MIC = 2), imipenem (MIC = 2), meropenem (MIC ≥32), trimethoprim/sulfamethoxazole (MIC >32), and aztreonam (MIC >256). Culture of the cervical lymph node biopsy sample grew *S. marcescens*.

After 7 months of treatment with ceftriaxone, clindamycin, and itraconazole, the patient completely recovered from the pneumonia and cervical lymphadenitis. She subsequently underwent successful hematopoietic stem cell transplant.

## Patient 2

In 2011, a 19-year-old white man from Ohio, USA, who had X-linked CGD was examined for right neck swelling and tenderness (2 weeks’ duration), a yellow ulcerated lesion on the right side of the hard palate, and an enlarged right tonsil with copious exudate. He had been receiving prophylactic trimethoprim-sulfamethoxazole and posaconazole. Erythrocyte sedimentation rate (ESR) was 28 mm/h, and C-reactive protein (CRP) concentration was 82 mg/L.

CGD had been diagnosed at birth on the basis of a positive family history. The patient had had hydrocephalus, catheter-associated fungal meningitis, and *Aspergillus fumigatus* pneumonia. When he was 15 years of age, CGD proctitis developed. Fourteen months before hospital admission, he had undergone right neck dissection for *Rothia aeria* infection, which was successfully treated with β-lactams (*8*).

CT images showed new bulky lymphadenopathy in the right neck, involving all nodal planes, and increased thickening and asymmetry of the right oropharynx with hypoattenuation of the right palatine tonsils. Culture of the right tonsillar exudate and empirical treatment with meropenem were not helpful. Antimicrobial therapy was switched to ceftriaxone and high-dose penicillin for empirical coverage of *G. bethesdensis* and *Actinomyces* spp. Right neck dissection with tonsillectomy yielded *Staphylococcus epidermidis*, and full 16S rRNA gene sequencing (≈1,500 bp) of 1 colony of a gram-negative bacillus showed a 99.8% match to the *G. bethesdensis* type strain. Nine weeks of vancomycin and ceftriaxone followed by 8 weeks of cefdinir, doxycycline, and rifampin led to complete resolution of the lymphadenitis.

## Patient 3

In 2012, a 16-year-old boy from Portugal who had X-linked CGD was examined for fever, cervical lymphadenopathy, and elevated inflammatory markers. CT images showed a deep cervical abscess, from which nothing grew on culture but which completely resolved after 5 weeks of intravenous ceftriaxone, doxycycline, and ciprofloxacin and 6 weeks of oral amoxicillin/clavulanate, ciprofloxacin, and doxycycline, along with prophylactic itraconazole and interferon-γ.

Immediately after completion of that course of antimicrobial drugs, pneumonia with pleural effusions developed. Results of all cultures (blood, lymph node, broncheoalveolar lavage, and pleural fluid) were negative, but full 16S rRNA gene sequencing (≈1,500 bp) of pleural fluid showed a >99% match to *Cupriavidus* spp. The patient received meropenem, ciprofloxacin, amikacin, doxycycline, teicoplanin, and voriconazole. Two weeks later, fever with splenomegaly, pancytopenia, low fibrinogen levels, and elevated ferritin and soluble CD25 levels were noted. Interferon-γ prophylaxis was discontinued and the patient was administered dexamethasone and intravenous immunoglobulin, after which the presumed exuberant inflammatory response quickly resolved. CT images of the neck and lung were unremarkable, as were positron emission tomography images. 

A month later, the boy was examined for fever, cough, and altered mental status; he required intubation and transfer to the intensive care unit. Magnetic resonance imaging revealed bilateral pneumonia and multiple intraparenchymal brain abscesses. Cerebrospinal fluid (CSF) was unremarkable. A lung biopsy sample, collected while the patient was receiving meropenem, ciprofloxacin, amikacin, doxycycline, teicoplanin, and voriconazole, was sterile. Voriconazole was switched to caspofungin and liposomal amphotericin B, and teicoplanin was switched to linezolid. The patient eventually recovered and was transferred out of the intensive care unit.

One month later, fever with focal neurologic deficits developed. CSF examination confirmed persistent pleocytosis with low glucose and elevated protein levels, and CT images indicated leptomeningitis. A 4-day culture of CSF on chocolate agar showed brownish colonies 1–2 mm in diameter. Full 16S rRNA gene sequencing (≈1,500 bp) performed on the isolate from CSF showed a 99.7% match to the *G. bethesdensis* type strain. Etest showed the following MICs (in μg/mL): tobramycin (MIC = 12), ceftriaxone (MIC >32), doxycycline (MIC = 24), and trimethoprim/sulfamethoxazole (MIC = 0.25). Isoniazid, clarithromycin, and rifampin had already been added to the patient’s treatment regimen. Despite the above interventions, the patient died of obstructive hydrocephalus and multiorgan failure.

## Patient 4

In 2013, a 9-year-old multiracial boy from Iowa, USA, who had X-linked CGD was examined for a 1-day history of fever, fatigue, decreased appetite, headache, and neck pain. He had been receiving oral trimethoprim/sulfamethoxazole, voriconazole and interferon-γ for CGD prophylaxis. When he was 1 month of age, he had had disseminated *Candida lusitaniae* infection with retropharyngeal, parapharyngeal, hepatic, and splenic abscesses. At 6 months of age, he was hospitalized for a progressively enlarging left posterior neck mass. A lymph node biopsy sample showed necrotizing granulomata and rare yeast forms suggestive of *Histoplasma*, but no specific organism was identified. He also had recurrent otitis media, tonsillitis, and aphthous stomatitis. Two months before the visit reported here, he had had *Aspergillus versicolor* pneumonia complicated by granulomatous appendicitis.

At the time of this hospital admission, he had 2 enlarged right anterior cervical nodes, which were soft, mobile, and not tender. ESR was 41 mm/h, and CRP concentration was 76 mg/L. CT images showed a 23 × 9 × 18-mm abscess adjacent to the right sternocleidomastoid muscle, extensive left supraclavicular lymphadenopathy, and left-sided pneumonia and pleural effusion.

Excisional biopsy of the right cervical lymph nodes yielded pus but no organisms. After 3 days of culture on chocolate agar, ≈20 tan colonies of an aerobic gram-negative bacillus were seen. The organism was oxidase-positive, catalase-positive, and indole-negative. After 6 days, abundant growth of a morphologically identical organism was seen on potato dextrose agar without antimicrobial agent and on Mycosel agar with chloramphenicol and cycloheximide but not on brain heart infusion agar with chloramphenicol and gentamicin (all media from Remel, Lenexa, KS, USA). Matrix-assisted laser desorption/ionization–time of flight mass spectrometry (Biotyper system version 3.1; Bruker Daltonics Inc., Billerica, MA, USA) from directly smeared colonies with and without formic acid overlay (*9*) yielded no identification, and growth was insufficient for biochemical identification or susceptibility testing. Species-level identification conducted by 16S rRNA gene sequencing (ABI MicroSeq 500 kit; Thermo Fisher Scientific, Grand Island, NY, USA, and the IDNS SmartGene system, version 3.6.10; SmartGene Inc., Raleigh, NC, USA) was interpreted as *A. methanolica* (100% identity >388 bp with type strain CGDAM1) (*7*). No other pathogens were grown or amplified from any specimen.

The patient initially received piperacillin/tazobactam and vancomycin; liposomal amphotericin B was administered in view of his recent *A. versicolor* pneumonia. After 4 days, the piperacillin/tazobactam was switched to meropenem and ciprofloxacin was added. Levels of inflammatory markers eventually returned to reference values, and the lymphadenopathy improved after 5 weeks of intravenous meropenem and intravenous and oral ciprofloxacin. The patient was discharged with ciprofloxacin, voriconazole for *A. versicolor* pneumonia, prophylactic trimethoprim/sulfamethoxazole, and interferon-γ. He subsequently underwent successful transplant of matched unrelated hematopoietic stem cells.

## Patient 5

In 2014, a 36-year-old white man from Georgia, USA, who had X-linked CGD was hospitalized with cervical and abdominal lymphadenopathy; he had 1-year history of fever, malaise, and weight loss. X-linked CGD had been diagnosed when the patient was 6 months of age, when he had had hepatosplenomegaly, cervical lymphadenitis, and recurrent *S. aureus* infections. *Aspergillus* spp. pneumonia developed when he was 2 years of age, a liver abscess required incision and drainage when he was 4 years of age, and multiple abdominal abscesses required surgery when he was 6 years of age. Prophylactic trimethoprim/sulfamethoxazole for CGD had been effective until the hospitalization reported here.

A year before admission, the patient had experienced sudden onset of fever, cough, and shortness of breath. Right-sided pleural effusion was treated with antimicrobial drugs and thoracentesis. Cough and dyspnea improved, but fever, fatigue, malaise, myalgia, and weight loss of >15 kg were refractory to hydroxychloroquine and interferon-γ. At 2 months before admission, he had had cervical and abdominal lymphadenopathy with ascites. Peritoneal fluid examination, esophagogastroduodenoscopy, and colonoscopy were not informative. Excisional biopsy of a right posterior cervical lymph node, tuberculin skin testing, and QuantiFERON–TB Gold (QIAGEN, Valencia, CA, USA) testing produced negative results. Ciprofloxacin and metronidazole did not abate symptoms and fever.

At admission, the patient had right supraclavicular and left axillary lymphadenopathy and hepatosplenomegaly. ESR was 88 mm/h, and CRP concentration was 131 mg/L. CT images showed lung scarring, splenomegaly, pericardial effusion, and multifocal adenopathy of the left axilla, mediastinum, celiac, periaortic, retroperitoneal, and mesenteric regions. Culture of excised axillary lymph node grew *S. epidermidis,* and *G. bethesdensis* (100% match to the *G. bethesdensis* type strain by full 16S rRNA gene sequencing, ≈1,500 bp). Etest of *G. bethesdensis* isolate showed the following MICs in μg/mL: tobramycin (MIC = 4), ceftriaxone (MIC = 32), doxycycline (MIC = 8), trimethoprim/sulfamethoxazole (MIC = 2), and tigecycline (MIC = 16).

The patient was empirically administered vancomycin and ceftriaxone and was discharged with ceftriaxone, doxycycline, prophylactic trimethoprim/sulfamethoxazole, and itraconazole. His medication was eventually switched to cefdinir along with CGD prophylaxis. After 15 months, inflammatory markers and left neck and supraclavicular lymphadenopathy had improved but had not yet normalized.

## Discussion

We have identified a total of 12 infections caused by 3 methylotroph bacteria in patients with CGD: 2 *A. methanolica*, 1 *M. lusitanum*, and 9 *G. bethesdensis* infections. Infections caused by *A. methanolica* and *G. bethesdensis* have been reported only for patients with CGD, whereas *M. lusitanum* in a patient without CGD undergoing chemotherapy for leukemia has been reported (*10*). These observations suggest that Nox2-based complex activity (superoxide production) is critical for protection against methylotroph infections. Consistent with this hypothesis, previous studies have demonstrated that *G. bethesdensis* persists in Nox2-based complex-deficient myeloid cells and is largely resistant to oxygen-independent microbicidal activity (*11**,**12*).

Methylotroph infections in CGD patients typically result in elevated inflammatory markers and lymphadenopathy, which may progress to necrotizing lymphadenitis with or without abscess formation. The clinical course may be protracted because of infection persistence, antimicrobial drug resistance, and relapse. Culturing these bacteria is difficult, requiring atypical media and prolonged incubation. These infections have not been fatal for patients in North and Central America, but *G. bethesdensis* infections in patients from Portugal and Spain (*5*) have caused fatal meningitis and bacteremia, respectively. The different clinical courses and outcomes for these patients compared with those in North and Central America suggest that the *G. bethesdensis* strain from Europe may be more virulent; animal studies are needed to explore this possibility. Furthermore, the strains isolated in Spain and Portugal showed only a 99.7% match with the 16S rRNA sequence of the type strain from North America, whereas most strains from North and Central America showed a 100% match with the type strain. Moreover, the strain from Europe displayed more in vitro resistance to antimicrobial agents and was resistant to colistin, most β-lactams, and quinolones (*5*). Although the value and accuracy of in vitro susceptibility testing for *G*. *bethesdensis* are unknown and may lack predictive value, the clinical and laboratory differences between *G*. *bethesdensis* strains from the United States and Europe may have substantial implications for therapy.

The facultative methylotrophs we report were difficult to culture; their correct identification required non–culture-based techniques, such as 16S rRNA gene sequencing, and a high index of suspicion. Application of 16S rRNA sequencing and molecular probes to target tissues may identify previously unrecognized bacteria, which may accompany and possibly facilitate methylotroph infections.

*G. bethesdensis* is a gram-negative, aerobic, oxidase-negative, and catalase positive bacillus. Culture is facilitated by specimen centrifugation and plating on buffered charcoal yeast extract agar or solid mycobacterial media; incubation takes up to 2 weeks (*6*). *A. methanolica* is a gram-negative, aerobic, acid-tolerant, catalase-positive, urease-positive, oxidase-positive, non–spore-forming, and nonmotile rod-shaped bacterium (*13*). It forms tan colonies in <5 days on chocolate agar and grows well on potato dextrose agar. *M. lusitanum* is a vacuolated gram-negative aerobic rod that is positive for indophenol oxidase, catalase, and urease and produces a pink pigment. It also reduces nitrate and assimilates malate (*10*). Methanol dehydrogenase serology is currently under investigation as a potential supportive diagnostic or prognostic tool for tracking methylotroph infections, particularly those caused by *G. bethesdensis* (*14*).

Management of methylotroph infections is often prolonged and may require combination antimicrobial drug therapy and surgery. Drug susceptibilities are difficult to determine and interpret. In vitro, *G. bethesdensis* is typically resistant to most penicillins, cephalosporins, carbapenems, and quinolones, but it is sometimes susceptible in vitro to ceftriaxone, aminoglycosides, doxycycline, and trimethoprim/sulfamethoxazole; combinations of these drugs have helped achieve initial resolution (*6*). Although antimicrobial drug susceptibilities for *A. methanolica* are not well defined, infections seemed to respond to combinations including meropenem, ciprofloxacin, gentamicin, doxycycline, and trimethoprim/sulfamethoxazole. *M. lusitanum* seems to be susceptible to aminoglycosides, cephalosporins, ciprofloxacin, piperacillin/tazobactam, and imipenem but not to meropenem, trimethoprim/sulfamethoxazole, or aztreonam. The role of surgery in treating methylotroph infections has not been defined, but its successful use in several cases is noteworthy.

In conclusion, methylotrophs are environmental organisms that can cause necrotizing infections in patients with CGD. Infectious prodromes and clinical courses may be prolonged. Diagnosis requires a high index of suspicion so that appropriate culture conditions and culture-independent techniques can be established for diagnosis. The difficulty of growing methylotrophs from infected lesions gives pause for the use of the label “sterile inflammation” with regard to CGD patients. Methylotrophs should be aggressively sought as the cause of chronic necrotizing infections in patients with CGD.
